# Basal Metabolic Rate and Body Composition Predict Habitual Food and Macronutrient Intakes: Gender Differences

**DOI:** 10.3390/nu11112653

**Published:** 2019-11-04

**Authors:** Xinyan Bi, Ciarán G. Forde, Ai Ting Goh, Christiani Jeyakumar Henry

**Affiliations:** 1 Clinical Nutrition Research Centre (CNRC)Singapore Institute for Clinical Sciences (SICS), Agency for Science, Technology and Research (A*STAR) and National University Health System Centre for Translational Medicine, Yong Loo Lin School of Medicine, National University of Singapore, Singapore 117599, Singapore; bi_xinyan@sics.a-star.edu.sg (X.B.); Ciaran_forde@sics.a-star.edu.sg (C.G.F.); Goh_Ai_Ting@sics.a-star.edu.sg (A.T.G.); 2 Department of Physiology, Medical Drive, MD 9 Building, National University of Singapore, Singapore 117593, Singapore; 3 Department of Biochemistry, Yong Loo Lin School of Medicine, National University of Singapore, Singapore 117599, Singapore

**Keywords:** basal metabolic rate, body composition, macronutrient intake, gender difference

## Abstract

The underlying mechanisms that regulate energy homeostasis and food intake are not fully understood. Moreover, little research has been performed on the relation of body composition with habitual macronutrient intake among free-living populations. Since body composition and energy metabolism differ between males and females, we aimed to determine whether the relationship between body composition and habitual macronutrient intakes is gender-dependent. In this cross-sectional study, 261 participants (99 males) were recruited from Singapore. Macronutrient intake was evaluated from a three-day self-reported dietary record. Body composition and basal metabolic rate (BMR) were determined by using dual-energy X-ray absorptiometry (DEXA) and indirect calorimetry, respectively. Our results show that both BMR (*p* < 0.001) and lean body mass (LBM, *p* < 0.001) predicted daily energy intake (EI). LBM was positively associated with intakes of protein (PRO) and fat (FAT) in females, but not in males. This relationship persisted even after adjustment for fat mass (FM). On the other hand, no significant associations between FM and macronutrient intake were observed in both males and females. Therefore, the relationship between habitual macronutrient intake, LBM, and BMR is gender-dependent. Elucidating the gender differences in energy metabolism is important for understanding the factors that regulate energy homeostasis and can subsequently help better manage energy balance.

## 1. Introduction

Increasing evidence suggested that unhealthy eating habits, including overeating and/or poor food selections, might be an important risk factor related to obesity development [1,2,3,4,5]. The concept that diet quality, or macronutrient intake adequacy, may affect weight change or body composition has been gaining momentum [5]. Although there are metabolic differences in protein (PRO), carbohydrate (CHO), or fat (FAT) intake on body weight regulation, no single dietary strategy or macronutrient composition has been shown to be superior for weight maintenance for the general population. Previous studies have demonstrated that low CHO and high PRO diets lead to more weight loss than high CHO and low FAT diets over three to six months [6,7,8]. However, other studies showed conflicting results [9,10]. Moreover, a long-term intervention did not demonstrate that low CHO and high PRO diets were superior to high CHO and low FAT diets [11,12,13,14]. In addition to influencing body weight per se, macronutrient intake may also influence body composition. Consumption of a low CHO and high FAT diet may induce loss of intra-abdominal adipose tissue during weight maintenance but may affect energy partitioning during weight loss [15]. Other studies have observed an inverse relationship between PRO intake and waist circumference (WC) [16]. Although total energy and macronutrient intake have been associated with obesity and body composition during weight loss or under controlled laboratory conditions, this has been less well studied among free-living populations. In addition, males and females distribute fat differently in the body. Therefore, it is necessary to examine whether gender affects the impact of macronutrient intake on body composition.

The observation that some individuals can maintain a relatively stable body weight over a long period of time suggests that energy intake (EI) is tightly linked with energy expenditure (EE) to maintain the energy balance [17]. Since an individual’s EE is closely related with their body composition and a large part of its variance is explained by lean body mass (LBM) or fat-free mass (FFM), body composition might play an important role in the drive to eat and, thus, to achieve an energy balance. A number of studies have shown that FFM, but not fat mass (FM), is a strong determinant of day-to-day food intake [18,19]. Moreover, Weise et al. [20] confirmed that FFM and 24-h EE were positively associated with ad-libitum food and individual macronutrient intake. They found that FFM-related EE increment must be compensated by increased EI via peripheral and/or central mechanisms. Regarding dietary causes of obesity, most studies have emphasized changing consumption patterns of FAT and CHO. In contrast, the role of protein has largely been ignored. Simpson et al. [21] have proposed a protein leverage hypothesis, which suggests that humans regulate their energy intake in terms of the protein consumed, including consuming more when required to maintain protein balance.

Taken together, these findings suggested that the macronutrient intake might affect the drive to eat, and, in turn, be influenced by an individual’s body composition. In this study, we examined the gender-dependent association of dietary intake of an individual macronutrient (CHO, FAT, and PRO) with body composition, including FM and LBM, as measured by dual-energy x-ray absorptiometry (DEXA) in 261 free-living people. We hypothesized that macronutrient intake is associated with the ratio between LBM or FM and that this relationship would be different between males and females.

## 2. Methods

### 2.1. Study Design

This study was a cross-sectional analysis of data from 261 participants attending a baseline visit from June 16, 2015 to November 24, 2016. The participants were recruited from the general public in Singapore. To be eligible, healthy participants (males and females) were required to be Singaporeans or Singapore permanent residents who have lived in Singapore for at least five years. Participants were excluded if they were pregnant, lactated, or took medicines. Prior to the study, all participants were asked to refrain themselves from intense physical activity as well as to restrict alcohol and caffeine-containing drinks. All procedures involving human subjects were approved by the National Healthcare Group Domain Specific Review Board (NHG DSRB, Reference Number: 2013/00783), Singapore. 

### 2.2. Macronutrient Intake

Dietary intake was assessed using three-day food diaries, based on estimates of household measures, on two weekdays and one weekend day [22]. On the test session day, subjects were briefed on how to fill in the diary with sample pictures and a sample of a recorded day included in the food diary template given to the subject to fill in. After receiving the food diary, the researcher would go through the food diary and call up the subject if any clarifications were required. Nutrient analysis was performed using the Food Works analysis program and FoodWorks 8.0.3553 (Xyris Software) nutrient databases with the local food database. The dietary outcomes examined were total energy, macronutrients (CHO, FAT, and PRO), sodium, potassium, fiber, and total sugars [22].

### 2.3. Anthropometry

Participants reached the laboratory in the morning after 10 hours of an overnight fast. All participants signed the informed consent form before starting. Body weight (kg) was measured to the nearest 0.1 kg by using an electrical scale. Height (cm) was measured to the nearest millimeter using a stadiometer (Seca 763 digital scale, Birmingham, United Kingdom). The smallest WC above the umbilicus or navel and below the xiphoid process was taken as WC (cm). All measurements were completed in duplicate and readings were averaged.

DEXA (QDR 4500A, fan-beam densitometer, Hologic, Waltham, USA, software version 8.21) was used for the measurement of FM and LBM. The basal metabolic rate (BMR) was measured using an indirect calorimeter (COSMED, Rome, Italy) in the fasting state. The participants lied in a supine position for 30 minutes without moving while a plastic canopy was placed over the upper part of their body. During the period of measurement, the participants were instructed to not sleep and to limit their movement. BMR was measured using the last 10 minutes of the measurement period to ensure stable and interpretable measurements are obtained.

### 2.4. Statistical Analysis

Statistical analysis was performed using the Statistical Package for the Social Sciences (SPSS) version 23. All data are expressed as means ± SD and checked for normality using the Shapiro-Wilk test. One-way ANOVA was used for between-group comparisons. Multivariate linear regression models were used to examine associations between BMR, body composition, i.e., FM and LBM with macronutrient intakes including PRO, FAT, and CHO. Partial correlations were used to determine the relationships between daily macronutrient intakes, LBM, and BMR. Two-sided *p* < 0.05 was considered statistically significant in all cases.

## 3. Results

Table 1 shows the baseline characteristics of the study population. Overall, there were significant differences in body composition including BMI (*p* = 0.001), WC (*p* < 0.001), PBF (*p* < 0.001), FM (*p* = 0.001), and LBM (*p* = 0.001) between males and females. With respect to diet composition, males had significantly higher daily macronutrient intakes, i.e., PRO (*p* = 0.001), FAT (*p* = 0.009), and CHO (*p* < 0.001) than females (Table 2). However, there were no significant differences between males and females in energy contribution of individual macronutrients. Among the 261 free-living participants studied, daily protein intake (1.23 g/kg/d), measured with respect to body weight, provided 16.7% of energy, while daily fat provided 28.3% (0.93 g/kg/d) and carbohydrate intake 53.0% (3.85 g/kg/d) of energy, respectively. Similarly, for Singaporean females, daily protein intake (1.31 g/kg/d) provided 17.1% of energy, while daily fat provided 29.2% (1.01 g/kg/d) of energy, and carbohydrate intake provided 51.6% (3.97 g/kg/d) of energy, respectively. According to the Singapore Health Promotion Board (HPB), the recommended PRO intake for an adult Singaporean is 1.07 g/kg/d [23]. For these participants, 39 males (39%) and 47 females (29%) had inadequate PRO intake.

**Figure 1:**
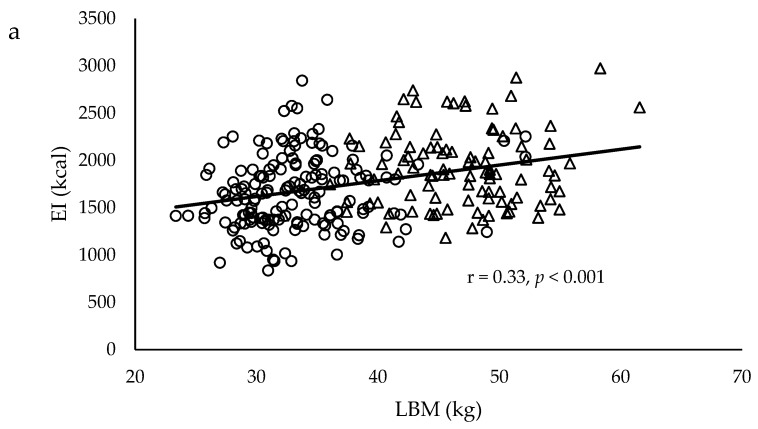

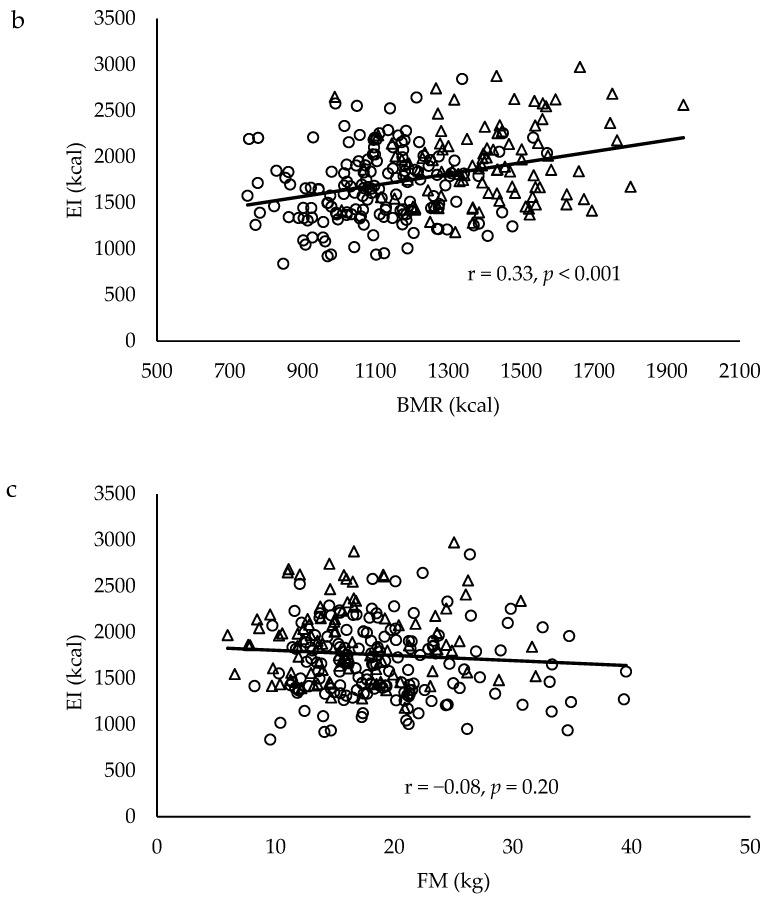
Scatter plots to illustrate the relationship between (**a**) LBM, (**b**) BMR, and (**c**) FM and daily EI. The relationships indicate significant correlation between LBM, BMR, and daily EI, but not FM. Males were represented by the open triangles and females were represented by the open circles.

Figure 1 shows the relationship between body composition, BMR, and daily EI. Correlation coefficients indicate significant positive associations between LBM (r = 0.33, *p* < 0.001), and BMR (r = 0.33, *p* < 0.001), and EI, but not FM (r = −0.08, p = 0.20). The association between LBM and EI remained significant after adjusting for age, smoking status, supplementary usage, family disease history, physical activity, and FM in females (*p* = 0.03), but not in males (*p* = 0.75, Table 3), which indicates the association was gender-dependent. In contrast, there were no significant associations between BMR and EI after adjusting for age and physical activity in both males and females (Table 4).

With respect to the individual macronutrients, after adjusting for age, smoking status, supplementary usage, family disease history, and physical activity levels, LBM was negatively associated with % CHO, regardless of gender (Table 3). In contrast, LBM was positively associated with PRO and FAT intakes in females, but not in males. Since high protein diets are usually associated with low carbohydrates, the observed association is likely due to decreased CHO intake as opposed to increased PRO intake. To further address the impact of CHO intake on the association between body composition and intakes of PRO and FAT, we repeated the analyses by adjusting CHO intake. All of the associations between dietary PRO, FAT, and LBM in females remained significant. Additionally, after adjusting for age, smoking status, supplementary usage, family disease history, physical activity levels, and LBM, there were no significant associations between FM and macronutrient intake in both males and females. LBM, on the other hand, when accounting for age, smoking status, supplementary usage, family disease history, physical activity levels, and FM, remained positively associated with PRO intake (*p* = 0.001), and FAT intake (*p* = 0.001), but not with CHO intake (Table 3). Table 4 shows that BMR was significantly associated with intake of PRO and FAT in females but not in males.

## 4. Discussion

The role of macronutrient intake in body building has been debated [9,24,25]. High PRO and low CHO diets are becoming increasingly popular and have been employed to evaluate the efficacy on weight loss and maintenance [7,26]. Several studies have shown that energy-restricted, high-PRO diets are more effective for weight loss compared to low-PRO diets [27,28,29,30], but other studies did not show any differences due to different PRO intakes [14]. However, most of the previous studies were conducted under energy restriction conditions where energy intake is curtailed to meet a weight loss target [14,27,28,29,30]. Due to the negative energy balance, an energy-restricted diet surely leads to weight loss. Some other weight loss maintenance studies demonstrated that diets with a modest increase in protein but a modest reduction in the glycemic index led to an improvement in the maintenance of weight loss [31]. So far, limited data is available regarding the roles of dietary macronutrient intake under normal free-living conditions with free access to food in the general population.

To the best of our knowledge, this is the first study to establish a relationship between habitual macronutrient intake and differences in the degree of FM and LBM between males and females. We found that the association of macronutrient intake with body composition was gender-dependent. The dietary pattern with high intake of PRO and FAT were correlated with high LBM in females, but not in males. Little evidence is available in the literature on potential underlying mechanisms to explain the different associations of body composition changes with habitual food intake between males and females. One possible explanation linked to LBM could be the combination of protein intake and physical activity [32]. However, in the current study, gender differences in intake were sustained even when controlling for participant physical activity. A previous population survey conducted in 14,000 American adults suggested that there were gender differences in eating habits [33]. While men were more likely to eat meat, women were more likely to eat fruits and vegetables. Therefore, increasing dietary PRO and FAT to exceed the minimum requirements may be a probable way to achieve a higher LBM profile in females. However, we did not analyze the eating patterns in our study due to the small sample size. Another explanation could be related to hormonal differences between genders. Unfortunately, data from the present study are insufficient to examine underlying mechanisms. 

Similar to previous studies [18,19,20,34], our results indicated that LBM predicts daily EI. However, in many of the previous studies, participant data was collected under controlled laboratory conditions, whereas, in the present study, participants consumed their normal diet while dietary EI and macronutrient composition varied as a function of food choice. These results suggest that individuals with higher LBM need more energy to preserve body mass [18] and LBM is a more powerful driver of daily EI. One possibility is that cytokins, such as IL-6, mysostatin, and IGF-1, which are released by the muscle tissue, may act as a powerful orexigenic signal to the central nervous system to control EI. Previously, lean tissue has been reported to act as an orexigenic feedback signal during periods of weight loss and weight regain [35,36]. Based on the results from semi-starvation studies, Dulloo et al. [35] noticed that, during significant weight loss, (e.g., >25% of body weight), FM and FFM losses independently predicted the post-starvation hyper-phagic response. Moreover, hyperphagia persisted until FFM was fully restored to pre-starvation levels despite the full restoration of body mass and FM. While the demands imposed by semi-starvation on body mass regulation clearly exceed those under free-living conditions, the idea of a physiological drive for food intake stemming from FFM is consistent with the “protein-stat” [37] and “amino-static” [38] theories of lean tissue and appetite regulation, respectively. Moreover, since LBM is highly correlated with BMR (r = 0.87, *p* < 0.001), the association between LBM and EI is possibly generated by the energy demand from LBM and reflected in BMR. In other words, the predominant contribution to our bodies’ energy expenditure is our LBM, and this, in turn, sets the minimum energy required to maintain the body’s lean tissues (including all vital organs), reflected in the minimum EI consumed at meals and over the whole day.

The strength of the current study includes that the potential confounding factors have been controlled for in all analyses. We controlled for both total EI and physical activity levels in all analyses since body composition is largely determined by EI and EE. We have shown that dietary PRO and FAT is positively associated with LBM determined by DEXA in a free-living population. Moreover, this association between dietary PRO, FAT, and LBM was gender-dependent. This suggests that it may be possible to achieve a more favorable LBM to FM ratio by increasing the amount of PRO and FAT in the diet of females, even in the absence of energy restriction. However, while there is a general lack of knowledge on how increases in LBM and BMR in females results in increased intake of PRO and FAT, there is no clear biological mechanism known that could explain the gender-dependent associations of LBM with food intake. 

## 5. Conclusions

In summary, we show that the positive associations between LBM, BMR, and dietary EI are gender-dependent, with a significant association between LBM and intake of PRO and FAT in females, but not in males. In this scenario, LBM-related increases in EE must be compensated by increased PRO and FAT intake via peripheral or central mechanisms. As a result, in the current energy-rich and nutrient-poor food environment, the drive to maintain LBM may promote increased energy intake and lead to overconsumption, especially among females.

## Figures and Tables

**Table 1:** Characteristics (mean ± standard deviation (SD)) of the study population.

Variable	Males		Females		*p* Value ^a^
	Mean ± SD	Range	Mean ± SD	Range	
*n*	99		162		
Age (year)	41.7 ± 14.6	21.0, 69.2	39.0 ± 14.6	21.0, 68.9	0.156
Height (cm)	170.4 ± 5.5	157.6, 184.3	159.6 ± 5.9	144.2, 174.8	<0.001
Weight (kg)	66.8 ± 9.5	45.4, 91.4	55.1 ± 9.7	34.5, 88.3	<0.001
BMI (kg/m^2^)	23.0 ± 3.0	16.3, 31.5	21.6 ± 3.5	16.1, 33.8	0.001
WC (cm)	78.7 ± 8.5	62.1, 104.3	69.9 ± 7.8	56.3, 96.1	<0.001
FM (kg) ^b^	16.5 ± 5.6	6.0, 31.9	19.0 ± 6.0	8.2, 39.5	0.001
LBM (kg) ^b^	46.8 ± 5.2	32.2, 61.6	33.2 ± 4.8	23.4, 52.2	<0.001
PBF (%) ^b^	24.7 ± 5.5	13.1, 37.2	34.5 ± 5.9	21.9, 51.1	<0.001

^a^ Gender differences by One-Way ANOVA. ^b^ Measured by DEXA. Abbreviations: BMI, body mass index. WC, waist circumference. PBF, percent body fat. FM, fat mass. LBM, lean body mass.

**Table 2:** Daily macronutrient intake, energy intake, and energy expenditure (mean ± SD and range) of the study population.

Variable	Males		Females		*p* Value ^a^
	Mean ± SD	Range	Mean ± SD	Range	
*n*	99		162		
PRO (g)	81.2 ± 29.1	32.2, 172.1	70.6 ± 21.5	20.2, 134.4	0.001
FAT (g)	61.2 ± 19.3	27.4, 121.8	54.6 ± 20.1	19.3, 155.5	0.009
CHO (g)	251.8 ± 48.7	169.5, 412.4	212.2 ± 53.8	72.0, 451.4	<0.001
EI (kcal)	1922.9 ± 405.0	1182.7, 2974.7	1657.7 ± 386.5	837.0, 2843.5	<0.001
RMR (kcal)	1403.9 ± 178.5	988.0, 1945.0	1093.5 ± 173.1	749.0, 1571.0	<0.001
% PRO	16.7 ± 3.9	8.8, 29.0	17.1 ± 3.8	8.2, 27.6	0.460
% FAT	28.3 ± 5.1	16.8, 41.8	29.2 ± 6.1	14.3, 54.9	0.224
% CHO	53.0 ± 6.5	34.2, 69.8	51.6 ± 7.9	27.5, 71.1	0.153
PRO (g/kg)	1.23 ± 0.46	0.48, 2.78	1.31 ± 0.42	0.43, 2.53	0.190
FAT (g/kg)	0.93 ± 0.31	0.43, 1.94	1.01 ± 0.37	0.35, 2.73	0.083
CHO (g/kg)	3.85 ± 0.94	2.00, 6.68	3.97 ± 1.18	1.22, 7.69	0.400

^a^ Gender differences by One-Way ANOVA. Abbreviations: PRO, mean daily protein intake. FAT, mean daily fat intake. CHO, mean daily carbohydrate intake. EI, mean daily energy intake. RMR, mean resting metabolic rate. % PRO, percentage of energy from protein. % FAT, percentage of energy from fat. % CHO, percentage of energy from carbohydrates.

**Table 3:** Sex-specific multivariable-adjusted regressions analysis for body composition variables with daily macronutrient intakes of the study population.

	Males (*n* = 99)	
	Model 1	Model 2
EI (kcal)	FM (kg): 0.04 (−12.77, 17.91) LBM (kg): 0.05 (−12.20, 19.30)	FM (kg): 0.02 (−16.58, 18.86) LBM (kg): 0.04 (−15.24, 21.18)
PRO (g)	FM (kg): 0.05 (−0.86, 1.37) LBM (kg): 0.10 (−0.62, 1.66)	FM (kg): 0.001 (−1.28, 1.28) LBM (kg): 0.10 (−0.80, 1.84)
FAT (g)	FM (kg): 0.09 (−0.47, 1.07) LBM (kg): 0.07 (−0.54, 1.04)	FM (kg): 0.07 (−0.65, 1.12) LBM (kg): 0.04 (−0.78, 1.05)
CHO (g)	FM (kg): −0.10 (−2.70, 0.96) LBM (kg): −0.10 (−2.76, 0.99)	FM (kg): −0.07 (−2.70, 1.52) LBM (kg): −0.07 (−2.76, 1.58)
%PRO	FM (kg): 0.04 (−0.13, 0.18) LBM (kg): 0.11 (−0.08, 0.24)	FM (kg): −0.03 (−0.20, 0.16) LBM (kg): 0.13 (−0.09, 0.28)
%FAT	FM (kg): 0.11 (−0.10, 0.32) LBM (kg): 0.11 (−0.10, 0.32)	FM (kg): 0.08 (−0.17, 0.31) LBM (kg): 0.07 (−0.17, 0.32)
%CHO	FM (kg): −0.20 (−0.49, 0.02) LBM (kg): −0.23 (−0.54, −0.02) *	FM (kg): −0.11 (−0.42, 0.16) LBM (kg): −0.18 (−0.52, 0.08)
Females (*n* = 162)		
EI (kcal)	FM (kg): −0.04 (−14.13, 8.23) LBM (kg): 0.12 (−4.35, 23.10)	FM (kg): −0.19 (−26.57, 1.60) LBM (kg): 0.24 (1.57, 36.33) *
PRO (g)	FM (kg): 0.04 (−0.47, 0.77) LBM (kg): 0.26 (0.40, 1.88) **	FM (kg): −0.19 (−1.46, 0.07) LBM (kg): 0.38 (0.74, 2.61) **
FAT (g)	FM (kg): 0.03 (−0.48, 0.70) LBM (kg): 0.24 (0.29, 1.70) *	FM (kg): −0.18 (−1.36, 0.09) LBM (kg): 0.36 (0.58, 2.37) **
CHO (g)	FM (kg): −0.12 (−2.69, 0.40) LBM (kg): −0.06 (-2.55, 1.28)	FM (kg): −0.14 (−3.32, 0.64) LBM (kg): 0.04 (−2.05, 2.83)
%PRO	FM (kg): 0.10 (−0.05, 0.17) LBM (kg): 0.25 (0.06, 0.32) *	FM (kg): −0.08 (−0.19, 0.09) LBM (kg): 0.30 (0.06, 0.40) *
%FAT	FM (kg): 0.08 (−0.09, 0.26) LBM (kg): 0.27 (0.12, 0.54) **	FM (kg): −0.12 (−0.35, 0.09) LBM (kg): 0.35 (−0.17, 0.70) **
%CHO	FM (kg): −0.11 (−0.38, 0.08) LBM (kg): −0.26 (−0.69, −0.14) **	FM (kg): 0.07 (−0.19, 0.37) LBM (kg): −0.30 (−0.83, −0.13) *

Model 1: adjustment for age, smoking status, supplementary usage, family disease history, and physical activity. Model 2: Model 1 added adjustment for FM and LBM. *p < 0.05. ** *p* < 0.005.

**Table 4:** Partial correlations between daily macronutrient intakes, LBM, and BMR of the study population ^a^.

Variables	Males (*n* = 99)		Females (*n* = 162)	
	LBM (kg)	BMR (kcal)	LBM (kg)	BMR (kcal)
PRO (g)	0.10	0.13	0.25 **	0.19 *
FAT (g)	0.07	0.15	0.23 *	0.19 *
CHO (g)	−0.10	−0.06	−0.05	0.07
EI (kcal)	0.05	0.13	0.11	0.15

^a^ Controlled for age, smoking status, supplementary usage, family disease history, and physical activity levels. * *p* < 0.05. ** *p* < 0.005.
